# SGLT2 inhibitors attenuate nephrin loss and enhance TGF-β_1_ secretion in type 2 diabetes patients with albuminuria: a randomized clinical trial

**DOI:** 10.1038/s41598-022-19988-7

**Published:** 2022-09-20

**Authors:** Yuan Tian, Xiao-min Chen, Xian-ming Liang, Xiao-bin Wu, Chun-meng Yao

**Affiliations:** 1 Department of Endocrinology and Metabolism, Zhongshan Hospital of Xiamen University, School of Medicine, Xiamen University, 201-209 Hubin South Road, Xiamen, 361004 People’s Republic of China; 2 Center of Clinical Laboratory, Zhongshan Hospital of Xiamen University, School of Medicine, Xiamen University, 201-209 Hubin South Road, Xiamen, 361004 People’s Republic of China; 3 Department of Nephrology, Zhongshan Hospital of Xiamen University, School of Medicine, Xiamen University, 201-209 Hubin South Road, Xiamen, 361004 People’s Republic of China

**Keywords:** Endocrine system and metabolic diseases, Chronic kidney disease

## Abstract

To evaluate the effect of SGLT2 inhibitor (SGLT2i) on albuminuria, nephrin (NPH) and transforming-growth-factor-beta_1_ (TGF-β1) levels in urine and low-grade inflammation in type 2 diabetes (T2D) patients. A randomized, blank-controlled clinical trial included 68 T2D patients and 10 controls. Based on the urinary albumin-to-creatinine ratio (UACR), 68 diabetic patients were stratified into three levels, UACR < 30 mg/g, UACR ≧ 30 mg/g to ≦ 300 mg/g and UACR ˃ 300 mg/g, who were randomized (1:1:1) to receive SGLT2i treatment for 12 weeks. The concentrations of NPH and TGF-β1 in urine were measured as indications of podocyte injury and renal fibrosis. Low-grade inflammation was assessed by the levels of IL-6, TNFα and hsCRP. After 12 weeks of SGLT2i treatment, the levels of UACR and NPH decreased, UTGF-β1 increased in the T2D with microalbuminuria and macroalbuminuria groups, NPH (1.12 [0.59, 1.29] vs. 0.71 [0.41, 1.07] µg/ml, *P* = 0.022) and (1.29 [0.99, 1.96] vs. 0.93 [0.57, 1.31] µg/ml, *P* = 0.002), UTGF-β1 (4.88 ± 1.31 vs. 7.27 ± 1.21 pg/ml, *P * < 0.001) and (4.30 ± 1.34 vs. 6.78 ± 2.59 pg/ml, *P * < 0.001), respectively. The changes in NPH were positively correlated with the UACR and negatively correlated with UTGF-β1 in T2D with albuminuria. SGLT2i alleviate nephrin loss and enhance TGF-β1 excretion in urine in T2DM with albuminuria. The anti-albuminuric effect of SGLT2i could be attributed to mitigating podocyte apoptosis and attenuating renal fibrosis.

*Trial registration* This clinical trial was registered on 15/10/2019, in ClinicalTrials.gov, and the registry number is NCT04127084.

## Introduction

Diabetic nephropathy (DN) is one of the most important chronic microvascular complications of type 2 diabetes and is also the most common cause of end-stage renal disease (ESRD) on a global scale. Early diagnosis and effective intervention for albuminuria are the core strategies to prevent the progression from DN to ESRD. The presence and deterioration of albuminuria and the increase in serum creatinine are still the main indicators for clinical evaluation of the progression of DN^1^. Therefore, effectively reducing albuminuria is extremely important and can reduce the risk of ESRD due to DN. Current standard albuminuria intervention strategies include sodium restriction, weight loss, blood pressure control, and inhibition or blocking of RASS activation^2^. However, these traditional methods did not take into account the effects of high sugar and high fat toxicity on local renal hemodynamics. Albuminuria may not be well controlled by simply following the above strategies. Sodium-glucose cotransporter 2 inhibitors (SGLT2i) are a newer class of hypoglycemic drugs that act on sodium-glucose cotransporter 2 in the curvature of the proximal tubule of the kidney, lower the renal glucose threshold, inhibit glucose and sodium reabsorption in proximal tubules, excrete excess urinary glucose and urinary sodium, transport urinary sodium to the distal convoluted tubule, stimulate tubuloglomerular feedback, contract afferent arterioles, and reduce the pressure of the glomerulus^3^. Therefore, SGLT2 inhibitors reduce glomerular hyperfiltration, effectively improve early glomerular hemodynamic abnormalities and reduce albuminuria. Animal experiments and human studies have shown that SGLT2i can reduce glucose, weight, and systolic blood pressure while reducing albuminuria^4–6^. Nevertheless, the mechanisms behind the renal protective effect of SGLT2 inhibitors are not fully understood. In the current trial, the concentrations of NPH and TGF-β1 in urine were measured as indications of podocyte injury and renal fibrosis. Low-grade inflammation was assessed by the levels of IL-6, TNFα and hsCRP. The purpose of our research was to observe the intervention of SGLT2i in type 2 diabetic patients with different levels of albuminuria for 12 weeks and to investigate the effect of SGLT2i on nephrin and TGF-β_1_ in urine, glucose and lipid metabolism, and low-grade inflammation in type 2 diabetes patients.

## Methods

### Participants

Based on the urinary albumin-to-creatinine ratio (UACR), type 2 diabetes patients were stratified into three levels, UACR < 30 mg/g, UACR ≧ 30 mg/g to ≦ 300 mg/g and UACR > 300 mg/g. Participants with T2D patients and healthy controls were allocated (2:2:2:1) to UACR < 30 mg/g, UACR ≧ 30 mg/g to ≦ 300 mg/g and UACR ˃ 300 mg/g or blank-controlled group. Considering the lost follow-up rate of at least 20%, 68 T2D patients and 10 normal controls were ultimately recruited between October 2019 and October 2020 at Zhongshan Hospital of Xiamen University in the Department of Endocrinology and Metabolism and Nephrology, which included 21 T2D with nonalbuminuria (UACR < 30 mg/g), 20 T2D with microalbuminuria (UACR30 ~ 300 mg/g), and 27 T2D with macroalbuminuria (UACR > 300 mg/g). Inclusion criteria for type 2 diabetes participants were as follows: the patients were initially diagnosed with type 2 diabetes according to World Health Organization (WHO) criteria, were 18–80 years of age, had HbA1c between 7 and 11%, received antidiabetic agents except SGLT2i and GLP-1, and had normal liver function and renal function. Exclusion criteria were set as follows: acute or chronic urinary tract infection, genital tract infection, type 1 diabetes, special type of diabetes, recent acute complications including diabetic ketoacidosis and hyperglycaemic hyperosmolar coma, acute or chronic glomerulonephritis, Long term use of corticosteroids or immunosuppressants, impaired liver function, impaired renal function (eGFR < 45 ml/min/1.73m^2^), women in pregnancy or lactation, alcoholics. The study was approved by the ethics committee of Zhongshan Hospital of Xiamen University and conducted in accordance with the Helsinki Declaration. Written informed consent was obtained from each participant.

### Research design and procedure

This was a 12-week, randomized, blank-controlled clinical trial included 68 T2D patients and 10 controls (trial registration *clinicaltrials.gov,* NCT04127084). In this clinical trial, 68 subjects with T2D were divided into three groups according to UACR levels: T2DM with nonalbuminuria (UACR < 30 mg/g), T2DM with microalbuminuria (UACR30 ~ 300 mg/g), and T2DM with macroalbuminuria (UACR > 300 mg/g), who were randomized (1:1:1) by computer-generated numbers to treated with dapagliflozin 10 mg per day (AstraZeneca, UK), empagliflozin 10 mg per day (EliLilly, USA) or canagliflozin 100 mg per day (Johnson & Johnson, USA) for 12 weeks. Considering the beneficial effects of RAS receptor blockers (ACEIs or ARBs) on albuminuria^7^, the subjects were asked to stop using ACEIs or ARBs for at least 4 weeks. If the patients’ SBP exceeded 140 mmHg or DBP exceeded 90 mmHg, CCB, β_1_ receptor blocker, and α receptor blocker were prescribed. We collected fasting blood and lean-catch 24-h urine samples for each participant before and after 12 weeks of treatment. All patients received standard care, including diet and exercise education, by professional nurses. The patients received follow-up every two weeks to take a routine urine test, measure blood glucose, systolic blood pressure (SBP), diastolic blood pressure (DBP), body mass index (BMI), body weight (BW), waist circumference (WC), hip circumference (HC), and WHR, and monitor the adverse events of drugs, including diabetic ketoacidosis, acute genital and urinary tract infection, bone fracture, leg and foot amputation, cancer.

### Primary and secondary endpoints

The primary endpoints were changes from baseline in UACR, NPH, UTGF-β_1_, IL-6, TNFα and hsCRP with SGLT2i treatment at week 12.

The secondary endpoints included changes from baseline in serum creatinine and eGFR, glucose and lipid metabolism with SGLT2i treatment at week 12.

### Clinical and laboratory measurement

BW, height, WC, HC. SBP and DBP were collected by professional nurses. BMI was calculated as the body weight in kilograms divided by the square of the patient’s height in meters. WC was measured midway between the lowest rib and the top of the iliac crest. HC was measured around the peak of the buttocks.

The waist-hip ratio (WHR) was calculated as the ratio of waist circumference to hip circumference. The enrolled patients fasted for at least 8 h before fasting cubital venous blood was drawn to determine HbA1c, FPG, Scr, UA, CRP, IL-6, and TNFα. On the other hand, 24 h of urine was collected to determine UACR, nephrin and TGF-β_1_. Patients’ renal and liver functions and plasma lipid and lipoprotein concentrations, including triglycerides (TG), total cholesterol (TC), low-density lipoprotein cholesterol (LDL-C), and high-density lipoprotein cholesterol (HDL-C) were assayed using standard methods (Roche cobas8000 automatic biochemical analyzer). The estimated glomerular filtration rate (eGFR) was based on the CKD-EPI creatinine equation^8^. Fasting blood glucose (FPG) was measured by the hexokinase method, high-sensitivity C-reactive protein (hsCRP) concentrations were measured using immune turbidimetry, uric acid (UA) was measured by the uricase-peroxidase method, and serum creatinine (Scr) was measured by the picric acid method. All measurements were performed using an automated method (Beckman Coulter Biochemical Analyzer, USA). Interleukin-6 (IL-6) and tumor necrosis factor α (TNFα) were measured by electrochemiluminescence (Roche Diagnostics, Switzerland). HbA1c was measured by high-performance liquid chromatography (HPLC) (Bio-Rad, Inc., Hercules, CA, USA). Urinary albumin was determined by immunoturbidimetry, and creatinine was measured by the picric acid method (Roche C701 automatic biochemical analyzer). Urinary TGF-β_1_ concentrations were assayed using a dual-antibody sandwich enzyme-linked immunosorbent assay (ELISA) kit (Multisciences, Hangzhou, China). Nephrin excretion was measured in the urinary samples of patients using quantitative ELISA kits in indirect competitive mode (Exocell, Inc., Philadelphia, USA).

### Statistical analysis

SPSS version 21 (IBM Inc., USA) and GraphPad Prism version 9.0 (Inc., La Jolla, CA, USA) were utilized for statistical analysis and the construction of graphs. Normally distributed data are presented as the mean±standard deviation. The skewed distribution variables are expressed as the median (interquartile range, IQR). The chi square test (*χ*^2^) was used to analyze the proportion of SGLT2i initiation, the percentage of metformin and DPP-IV inhibitors in baseline medications in this study. The comparisons of basic characteristics among multiple groups were made using one-way analysis of variance. Comparisons of baseline and posttreatment data in the same group were made using a paired* t* test or Wilcoxon signed rank test. The Wilcoxon signed rank test was used to determine the changes in NPH, TGF-β_1_, hsCRP, IL-6 and TNFα before and after 12 weeks of SGLT2i treatment. Spearman correlation coefficients were calculated to explore the relationships between baseline UACR and NPH, eGFR, Scr, Age, HbA1c, SBP, hsCRP, and IL-6, to explore the relationship between changes in urinary nephrin and those in urinary TGFβ_1_, UACR, eGFR, and weight before and after 12 weeks of treatment with SGLT2i in type 2 diabetes with albuminuria. Two-tailed significance was set at *P* < 0.05.

## Results

### Comparisons of baseline characteristics of the study participants

The clinical and laboratory characteristics are shown in Table 1. Sex, TC, TG, HDL-C, UA, UTGF-β_1_ and TNFα were comparable between the four groups. As expected, patients with T2D had higher age, BW, BMI, WC, WHR and HbA1c than normal controls. In the baseline analysis, participants’ duration, BW, WC, HC, WHR, HbA1c, SBP, DBP, hsCRP, and IL-6 among the three diabetic groups showed no statistically significant differences. Patients with lower baseline UACR categories (UACR < 30 mg/g) were more likely to be younger and have a higher baseline BMI. There were significant differences in the values of serum creatinine (Scr) and LDL-C in all four groups (*P* < 0.05), as shown in Table 1.

**Table 1:** Comparisons of baseline characteristics in study participants.

Variable	NC	UACR < 30 mg/g	UACR $\geqslant$ 30 to $\leqslant$ 300 mg/g	UACR > 300 mg/g	*P*-value
	n = 10	n = 21	n = 20	n = 27	
Age (years)	45 ± 17^c^	45 ± 13^c^	59 ± 13^ab^	58 ± 12^ab^	0.001
Male/Female (n)	6/4	13/8	12/8	16/11	0.998
BW (kg)	61.15 ± 8.01^b^	76.50 ± 12.67^a^	72.33 ± 16.07^a^	68.63 ± 14.58	0.012
BMI (kg/m^2^)	23.04 ± 3.10^bc^	31.72 ± 3.22^ac^	28.31 ± 4.13^ab^	26.06 ± 4.27^ab^	< 0.001
WC (cm)	76 ± 8^b^	88 ± 9^a^	91 ± 11^a^	87 ± 11^a^	0.003
HC (cm)	90 ± 5	97 ± 6^a^	98 ± 10^a^	94 ± 8	0.003
WHR	0.85 ± 0.06	0.91 ± 0.05^a^	0.93 ± 0.07^a^	0.93 ± 0.06^a^	0.003
Duration (year)	NA	4 (2, 8)	8 (4, 11)	8 (4, 11)	0.071
*Medicine (%)*					
Metformin	NA	12/21 (57%)	9/20 (45%)	14/27 (52%)	0.738
DPP-IV inhibitor	NA	6/21 (29%)	5/20 (25%)	7/27 (26%)	0.964
SBP (mmHg)	116 ± 13	128 ± 14	135 ± 17^a^	138 ± 17^a^	0.002
DBP (mmHg)	66 ± 8	79 ± 12^a^	79 ± 11^a^	78 ± 9^a^	0.010
TC (mmol/L)	4.85 ± 0.68	5.06 ± 1.03	4.36 ± 0.78	5.20 ± 1.32	0.100
TG (mmol/L)	1.21 (0.65, 1.55)	1.54 (1.08, 2.31)	1.69 (1.20, 2.03)	1.34 (1.17, 2.45)	0.311
LDL-C (mmol/L)	3.05 ± 0.66	3.21 ± 0.73	2.67 ± 0.59^b^	3.34 ± 0.86^c^	0.036
HDL-C (mmol/L)	1.38 ± 0.40	1.13 ± 0.16	1.21 ± 0.29	1.15 ± 0.23	0.311
HbA1c (%)	5.38 ± 0.41	7.53 ± 1.41^a^	8.31 ± 1.38^a^	7.90 ± 1.78^a^	< 0.001
Scr (umol/L)	67 ± 15	78 ± 14	74 ± 16	99 ± 42^ac^	0.035
UA (umol/L)	340 ± 92	358 ± 100	347 ± 101	395 ± 87	0.329
eGFR (ml/min/1.73m^2^)	109 ± 22	99 ± 21	88 ± 20	74 ± 24^ab^	< 0.001
UACR (mg/g)	3 (1, 4)	6 (4, 12)	87 (42, 203)^ab^	861 (459, 1966)^abc^	< 0.001
NPH (µg/ml)	1.21 (0.36, 1.68)	0.75 (0.46, 1.26)	1.12 (0.59, 1.29)	1.29 (0.99, 1.96)^b^	0.043
UTGFβ1 (pg/ml)	3.61 ± 0.84	4.34 ± 1.52	4.88 ± 1.31	4.30 ± 1.34	0.078
hsCRP (mg/L)	0.76 (0.26, 1.24)	1.69 (0.69, 3.59)	1.69 (0.88, 3.68)	2.39 (0.96, 4.55)^a^	0.028
IL-6 (pg/ml)	1.50 (1.50, 3.95)	2.43 (1.50, 4.90)	2.85 (1.58, 4.50)	4.31 (2.30, 6.30)^a^	0.045
TNFα (pg/ml)	12.3 (6.35, 35.23)	14.50 (7.34, 25.80)	23.2 (12.68, 65.25)	27.60 (9.57, 180)	0.121

Data are expressed as mean ± standard deviation or median (interquartile rang).

*NC* normal control, *BW* body weight, *BMI* body mass index, *WC* Waist Circumference, *HC* Hip Circumference, *WHR* Waist hip ratio, *SBP* Systolic blood pressure, *DBP* Diatolic blood pressure, *TC* Total choles-terol, *TG* triglycerides, *LDL-C* low-density lipoprotein cholesterol, *HDL-C* high-density lipoprotein cholesterol, *HbA1c* glycated haemoglobin, *Scr* serum creatinine, *UA* Uric acid, *eGFR* estimated Glomerular Filtration Rate, *UACR* urine albumin/creatinine, *UTGFβ1* urine transforming-growth-factor-beta1, *NPH* nephrin, *hsCRP* High sensitivity C-reactive protein, *IL-6* Interleukin-6, *TNFα* Tumor necrosis factor α.

^a^Versus NC Group, *P* < 0.05.

^b^Versus UACR < 30 mg/g Group, *P* < 0.05.

^c^Versus UACR ≥ 30to ≤ 300 mg/g Group, *P* < 0.05.

The percentage of metformin and DPP-IV inhibitors in the baseline analysis were not statistically significant between different UACR levels, as shown in Table 1.

Three SGLT2 inhibitors, including dapagliflozin, empagliflozin, and canagliflozin, of which 22 (32.4%) used dapagliflozin, 27 (39.7%) used empagliflozin, and 19 (27.9%) used canagliflozin, were randomized to administer throughout the study. The percentage of SGLT2 inhibitor initiation was not statistically significant, as shown in Table S1.

### SGLT2 inhibitor treatment decreased UACR and nephrin levels

The median values of baseline UACR (6, 87, 861 mg/g, *P* < 0.001) gradually increased in the three diabetic groups. Patients with higher baseline UACR (UACR ≥ 300 mg/g) categories were more likely to have lower eGFR and higher Scr (*P* < 0.05). Spearman rank correlations showed that NPH (*r* = 0.317, *P* = 0.005), eGFR (*r* = − 0.558, *P* < 0.001), Scr (*r* = 0.299, *P* = 0.009), age (*r* = 0.398, *P* < 0.001), HbA1c (*r* = 0.340, *P* = 0.003), SBP (*r* = 0.415, *P* < 0.001), hsCRP (*r* = 0.296, *P* = 0.009) and IL-6 (*r* = 0.260, *P* = 0.023) were highly relevant to the baseline UACR, as shown in Table S2.

**Figure 1:**
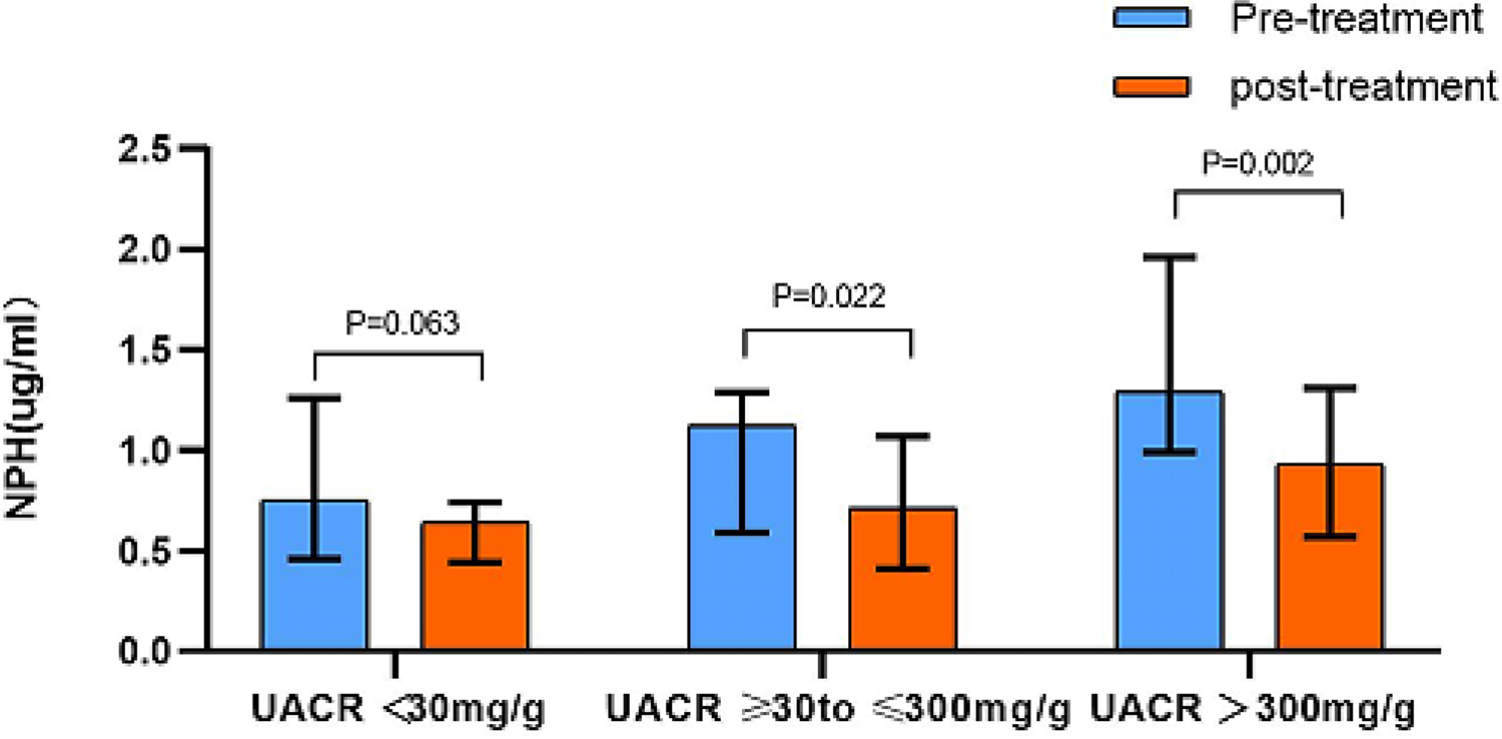
Changes of nephrin (NPH) before and after 12-week treatment with SGLT2 inhibitors in different UACR levels. *NPH* nephrin, *UACR* urinary albumin-to-creatinine ratio.

The median values of baseline nephrin (0.75, 1.12, 1.29 µg/mL, *P* = 0.014) showed significant differences among the three diabetic groups, in which nephrin and Scr were highest and GFR was lowest at baseline UACR ≥ 300 mg/g. After 12 weeks of SGLT2 inhibitor treatment, the UACR decreased in the microalbuminuria and macroalbuminuria groups compared to baseline, which were 87 [42, 203] vs. 75 [21, 152] mg/g, *P* = 0.003 and 861 [459, 1966] vs. 738 [213, 1482] mg/g, *P* = 0.037, respectively. The values of NPH were significantly lower in the microalbuminuria and macroalbuminuria groups than at baseline (1.12 [0.59, 1.29] vs. 0.71 [0.41, 1.07] µg/ml, *P* = 0.022 and 1.29 [0.99, 1.96] vs. 0.93 [0.57, 1.31] µg/ml, *P* = 0.002, respectively) (Fig. 1, Table 2).

**Table 2:** Comparisons of parameters before and after 12- week treatment with SGLT2 inhibitors in type 2 diabetes.

Variable	UACR < 30 mg/g			UACR $\geqslant$ 30 to $\leqslant$ 300 mg/g			UACR > 300 mg/g		
	Pre-treatment	Post-treatment	*P*-value	Pre-treatment	post-treatment	*P*-value	Pre-treatment	Post-treatment	*P*-value
BW (kg)	76.50 ± 12.67	73.04 ± 12.55	< 0.001	72.33 ± 16.07	69.50 ± 15.39	< 0.001	68.63 ± 14.58	65.84 ± 14.12	< 0.001
BMI (kg/m^2^)	31.72 ± 3.22	25.69 ± 3.66	< 0.001	28.31 ± 4.13	26.92 ± 3.65	< 0.001	26.06 ± 4.27	24.98 ± 4.02	< 0.001
WC (cm)	88 ± 9	84 ± 8	< 0.001	91 ± 11	87 ± 10	< 0.001	87 ± 11	84 ± 11	< 0.001
HC (cm)	97 ± 6	95 ± 7	0.068	98 ± 10	95 ± 8	0.001	94 ± 8	91 ± 7	< 0.001
WHR	0.91 ± 0.05	0.88 ± 0.04	0.003	0.93 ± 0.07	0.91 ± 0.06	0.014	0.93 ± 0.06	0.92 ± 0.07	0.226
SBP (mmHg)	128 ± 14	123 ± 14	0.148	135 ± 17	130 ± 15	0.146	138 ± 17	134 ± 15	0.126
DBP (mmHg)	79 ± 12	77 ± 12	0.258	79 ± 11	73 ± 10	0.018	78 ± 9	76 ± 11	0.506
TC (mmol/L)	5.06 ± 1.03	5.03 ± 0.94	0.894	4.36 ± 0.78	4.49 ± 1.09	0.48	5.20 ± 1.32	4.91 ± 1.13	0.269
TG (mmol/L)	1.54 (1.08, 2.31)	1.35 (1.01, 2.05)	0.191	1.69 (1.20, 2.03)	1.41 (0.92, 1.93)	0.028	1.34 (1.17, 2.45)	1.62 (1.13, 2.64)	0.638
LDL-C (mmol/L)	3.21 ± 0.73	3.33 ± 0.74	0.476	2.67 ± 0.59	2.85 ± 0.82	0.118	3.34 ± 0.86	3.21 ± 0.84	0.433
HDL-C (mmol/L)	1.13 ± 0.16	1.18 ± 0.13	0.021	1.21 ± 0.29	1.24 ± 0.24	0.32	1.15 ± 0.23	1.17 ± 0.24	0.493
HbA1c (%)	7.53 ± 1.41	7.12 ± 1.12	0.054	8.31 ± 1.38	7.46 ± 1.16	0.042	7.90 ± 1.78	7.43 ± 1.27	0.15
Scr (umol/L)	78 ± 14	74 ± 14	0.137	74 ± 16	73 ± 18	0.92	99 ± 42	100 ± 39	0.563
UA (umol/L)	358 ± 100	327 ± 86	0.048	347 ± 101	367 ± 103	0.481	395 ± 87	372 ± 70	0.156
eGFR (ml/min/1.73m^2^)	99 ± 21	103 ± 19	0.156	88 ± 20	87 ± 22	0.764	74 ± 24	70 ± 21	0.09
UACR (mg/g)	6 (4, 12)	6 (4, 10)	0.848	87 (42, 203)	75 (21, 152)	0.003	861 (459, 1966)	738 (213, 1482)	0.037
NPH (µg/ml)	0.75 (0.46, 1.26)	0.64 (0.44, 0.74)	0.063	1.12 (0.59, 1.29)	0.71 (0.41, 1.07)	0.022	1.29 (0.99, 1.96)	0.93 (0.57, 1.31)	0.002
UTGFβ1 (pg/ml)	4.34 ± 1.52	6.78 ± 1.36	< 0.001	4.88 ± 1.31	7.27 ± 1.21	< 0.001	4.30 ± 1.34	6.78 ± 2.59	< 0.001
hsCRP (mg/L)	1.69 (0.69, 3.59)	1.57 (0.85, 3.59)	0.52	1.69 (0.88, 3.68)	1.72 (0.81, 2.23)	0.36	2.39 (0.96, 4.55)	2.04 (0.96, 3.34)	0.517
IL-6 (pg/ml)	2.43 (1.50, 4.90)	3.06 (1.50, 4.06)	0.913	2.85 (1.58, 4.50)	2.85 (1.96, 5.05)	0.856	4.31 (2.30, 6.30)	3.48 (2.46, 5.23)	0.534
TNFα (pg/ml)	14.5 (7.34, 25.80)	9.96 (5.15, 116)	0.590	23.2 (12.68, 65.25)	17 (8.35, 79.75)	0.446	27.6 (9.57, 180)	26.1 (7.67, 142)	0.564

Data are expressed as mean ± standard deviation or median (interquartile rang).

*BW* body weight, *BMI* body mass index, *WC* Waist Circumference, *HC* Hip Circumference, *WHR* Waist hip ratio, *SBP* Systolic blood pressure, *DBP* Diatolic blood pressure, *TC* Total cholesterol, *TG* triglycerides, *LDL-C* low density lipoprotein cholesterol, *HDL-C* high density lipoprotein cholesterol, *HbA*_*1*_*c* glycated haemoglobin, *Scr* serum creatinine, *UA* Uric acid, *eGFR* estimated Glomerular Filtration, *UACR* urine albumin-to-creatinine ratio, *NPH* nephrin, *UTGFβ1* urine transforming-growth-factor-beta1, *hsCRP* High sensitivity C reactive protein, *IL-6* Interleukin 6, *TNFα* Tumor necrosis factor α.

### SGLT2 inhibitor treatment dramatically enhanced TGF-β_1_ secretion

**Figure 2:**
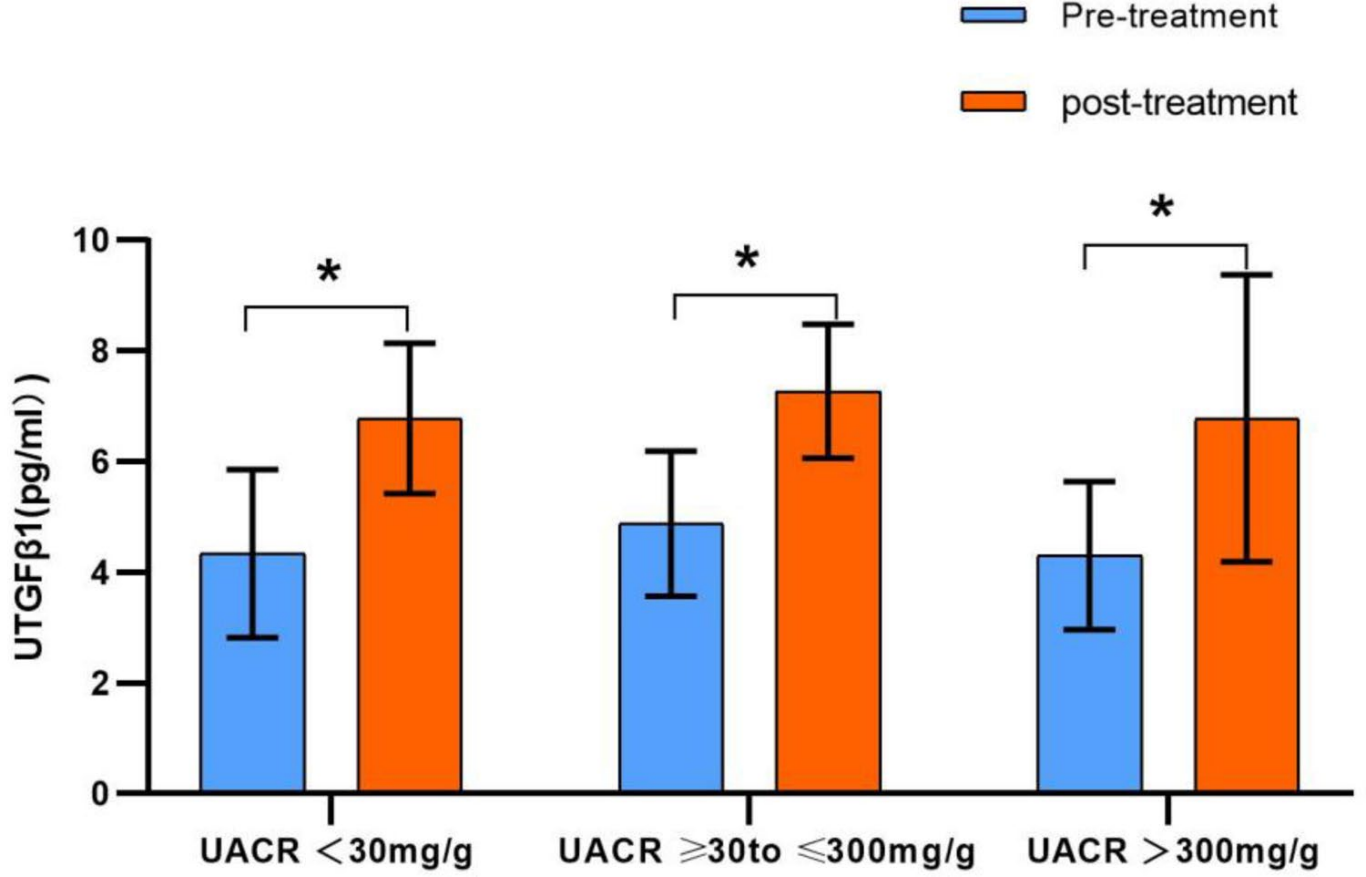
Changes of UTGFβ1 before and after 12-week treatment with SGLT2 inhibitors in different UACR levels. *UTGFβ1* urine transforming-growth-factor-beta1, *UACR* urinary albumin-to-creatinine ratio, *P < 0.001.

We found that the mean values of baseline UTGF-β_1_ (4.34, 4.88, 4.30 pg/ml) were comparable across the three diabetic groups. After 12 weeks of SGLT2i treatment, TGF-β_1_ secretion in urine was significantly enhanced in the three groups compared with baseline, which was 4.34 ± 1.52 versus 6.78 ± 1.36 pg/mL, *P* < 0.001, 4.88 ± 1.31 versus 7.27 ± 1.21 pg/mL, *P* < 0.001 and 4.30 ± 1.34 versus 6.78 ± 2.59 pg/mL, *P* < 0.001, respectively (Fig. 2, Table 2).

### SGLT2i treatment changed glucose and lipid metabolism, inflammation and weight loss

After 12 weeks of SGLT2i treatment, BW, BMI, and WC were remarkably reduced in the three diabetic groups regardless of the baseline UACR (all *P * < 0.001). The values of UA (358 ± 100 vs. 327 ± 86 µmol/L, *P* = 0.048) and WHR (0.91 ± 0.05 vs. 0.88 ± 0.04, *P* = 0.003) were significantly lowered, and HDL-C was significantly increased (1.13 ± 0.16 vs. 1.18 ± 0.13 mmol/L, *P* = 0.021) compared with baseline in the UACR < 30 mg/g group, while TG, HbA1c, HC, WHR and DBP were markedly reduced in the microalbuminuria group after 12 weeks of SGLT2i treatment (all *P*  <  0.05) (shown in Table 2).

After 12 weeks of SGLT2i treatment, the levels of TNFα, hsCRP, and IL-6 showed no statistically significant differences compared with baseline among the three diabetic groups (shown in Table 2).

### Correlations between urinary nephrin and other variables

Spearman’s correlation analysis showed that the changes in NPH were positively correlated with the UACR (*r* = 0.287, *P* = 0.006), which was negatively correlated with UTGF-β1 (*r* = − 0.373, *P * < 0.001), eGFR (*r* = − 0.224, *P* = 0.034), BMI (*r* = − 0.376, *P * < 0.001), WC (*r* = − 0.356, *P * < 0.001), WHR (*r* = − 0.248, *P* = 0.018) after 12 weeks of SGLT2i treatment in T2D patients with albuminuria.

## Discussion

SGLT2i have been demonstrated to exert an important impact on renal composite outcomes, including reducing albuminuria by 30–40% in large-scale clinical trials^9,10^. The increased albuminuria is an indicator of glomerular injury with loss of glomerular permselectivity. The first stages of DN are characterized by microalbuminuria and a small increase in albumin excretion in urine. Later stages are defined by macroalbuminuria or proteinuria leading to a decreased glomerular filtration rate^11^. Our study demonstrated that type 2 diabetic patients with a higher baseline UACR, namely, macroalbuminuria categories, were more likely to have a lower eGFR and higher Scr. After 12 weeks of SGLT2i treatment, the UACR markedly decreased in the microalbuminuria and macroalbuminuria groups compared to baseline. However, the precise mechanism by which SGLT2 inhibitors reduce albuminuria in diabetic neuropathy is not fully understood.

Our findings show that baseline UACR was positively correlated with nephrin expression. After 12 weeks of SGLT2i treatment, UACR and NPH remarkably decreased in the T2D with microalbuminuria and macroalbuminuria groups. The pathogenesis of albuminuria in diabetic nethropathy has been linked to podocyte foot process effacement (FPE), reduced nephrin-positive areas in glomeruli, mesangial expansion, glomerular basement membrane thickening, and increased area of TGF-β1 staining in glomeruli, which result in podocyte apoptosis, tubulointerstitial collagen accumulation, glomerular fibrosis and glomerulosclerosis. Therefore, podocyte apoptosis plays a central and critical role in the disruption of the structural and functional integrity of the glomerular filtration barrier^12^. Nephrin is a single-pass transmembrane receptor molecule located at the specialized podocyte cell–cell junction termed the slit diaphragm (SD). The slit diaphragm is the final barrier preventing passage of proteins into the urinary filtrate. Mutations or downregulation of nephrin is associated with loss of defective filtration barrier, causing albuminuria^13^. Our results indicated that baseline nephrin in urine gradually increased with the progression of albuminuria, nephrin loss into urine was highest and eGFR was slowest in the macroalbuminuria subgroup.

Nephrin, a podocyte marker, is not only an essential protein in the SD complex but also involved in podocyte survival. Its extracellular domain forms the SD zipper-like structure, while its intracellular domain acts as a signaling molecule^14^. Our research showed that the change in NPH was positively correlated with the UACR and negatively correlated with UTGF-β1 after 12 weeks of SGLT2i treatment in T2D patients with albuminuria. Nephrin loss is a sensitive marker of podocyte apoptosis. Common antidiabetic agent, metformin and DPP-4 inhibitors, can also ameliorate podocyte damage by restoring renal tissue nephrin expression^15,16^. We have compared the application of metformin and DPP-IVinhibitors between different groups in the baseline analysis to eliminate their effects on the nephrin expression. Our result showed that the percentage of metformin and DPP-IV inhibitors between different UACR levels groups were not statistically significant. Staining for the presence of nephrin showed a significant decrease in Western diet-fed C57BL/6J mice compared with Low fat diet-fed mice. Dapagliflozin prevented loss of podocytes as determined by Immunofluorescence staining for nephrin^17^.

TGF-β1, a potent fibrogenic cytokine, inhibits the expression of the slit-diaphragm protein nephrin and results in podocyte apoptosis in several ways. First, TGF-β1 induces podocyte apoptosis by activating caspase3 via p38 mitogen-activated protein kinase (MAPK) and Smad7. In TGF-β1 transgenic mice, TGF-β induces apoptosis by activating p38MAPK and the classic effector caspase-3^18^. Dapagliflozin prevented glomerular pathology and renal fibrosis in Western diet -fed mice, which could relieve the increases in extracellular matrix accumulation in the tubular interstitium as determined by type IV collagen and fibronectin, mitigate mesangial expansion, reduce albuminuria^17^. Second, TGFβ1 but not HG increased SGLT2 expression via phosphorylated smad3 and inhibited cell survival signaling NF-κB, resulting in amplification of TGF-β–mediated podocyte apoptosis^19^. Canagliflozin (CAN) transcriptionally inhibited NLRP3 inflammasome-related proteins by inhibiting the transduction of the nuclear factor κB (NFκB) signal^20^. Third, TGF-β1 is known to activate the Notch pathway^21,22^, which is correlated with albuminuria and glomeruloesclerosis^23^. The Notch activation induced by TGF-β1 has been involved in epithelial mesenchymal transition, augmented vascular endothelial growth factor (VEGF) expression, decreased nephrin expression and podocyte number. Blockade of Notch-1 signaling significantly abrogated VEGF activation and nephrin repression in HG-stressed cells and ameliorated albuminuria in the diabetic kidney^24^. SGLT2 inhibitors blocked TGF-β1-induced mRNA expression of thrombospondin 1 (THBS1), tenascin C (TNC), and platelet-derived growth factor subunit B (PDGF-B), which are key mediators of renal fibrosis and kidney disease progression in two human proximal tubular (PT) cell lines^25^. Dapagliflozin significantly reduced effluent TGF-β concentrations, peritoneal thickening and fibrosis, resulting in improved ultrafiltration in a mouse model of chronic peritoneal exposure to high-glucose dialysate^26^. Empagliflozin treatment or downregulation of SGLT-2 expression significantly ameliorated peritoneal fibrosis by suppressing TGF-β/Smad signaling-associated proteins, such as TGF-β1 and phosphorylated Smad3 (p-Smad3)^27^.

We have demonstrated for the first time that SGLT2 inhibitors alleviate nephrin loss and enhance TGF-β1 excretion in urine at the same time in type 2 diabetic patients with albuminuria. This means that administration of SGLT2 inhibitors in diabetic nethropathy could restore nephrin expression in glomeruli, attenuate nephrin loss in the urine and relieve podocyte apoptosis, inhibit TGF-β1 expression in glomeruli, enhance TGF-β1 secretion by the renal pathway, and ameliorate the progression of renal fibrosis, which is aligned with the results in db/db diabetic mice^28^. Therefore, SGLT2i should be considered one of the most promising drugs to mitigate podocyte apoptosis and alleviate renal fibrosis.

Significant body weight loss from baseline was observed after 12 weeks of SGLT2i treatment, which caused a mean weight loss of approximately 2–3 kg regardless of the baseline UACR. Furthermore, Spearman’s correlation analysis showed that the changes in NPH were negatively correlated with BMI, WC, and WHR after 12 weeks of SGLT2i treatment in T2D patients with albuminuria. Obesity is associated with an increased risk of CKD. There are potential mechanisms that can explain the association between obesity and renal injury, including hemodynamic effects, inflammation and renal lipotoxicity^29, 30^. SGLT2i treatment had a beneficial effect on weight loss, which was attributed to improved primary glucose transporters in proximal tubular cells (PTCs), blocked reabsorption of filtered glucose, and led to massive glycosuria and deprivation of energy. The decrease in body weight is the result of two major effects of SGLT2 inhibition: caloric loss due to glucose excretion and loss of body water due to osmotic diuresis, which reduce glomerular pressure and inhibit albuminuria excretion^31,32^.

The strengths of this study are that it is the first to verify that SGLT2 inhibitors alleviate nephrin loss and enhance TGF-β1 secretion in urine at the same time in type 2 diabetic patients with albuminuria. Moreover. The changes in NPH were negatively correlated with UTGF-β1 after 12 weeks of SGLT2i treatment in T2D with albuminuria. However, this study has certain limitations. First, this was a randomized, blank-controlled clinical trial study with a relatively small sample size and a comparatively short study period, lack of active-controlled group. Second, further studies are needed to explore the internal mechanisms by which SGLT2i attenuate podocyte apoptosis and reduce the secretion of nephrin with respect to changes in glomerular ultrastructure, inflammatory pathway transcription and translation via histological results of renal biopsy.

## Conclusion

In conclusion, this study demonstrated that SGLT2 inhibitors can reduce albuminuria, decrease nephrin loss and enhance TGF-β1 secretion in urine. Furthermore, the changes in NPH were positively correlated with the UACR and negatively correlated with UTGF-β1 after 12 weeks of SGLT2i treatment in T2D patients with albuminuria. Significant weight loss was also observed after 12 weeks of SGLT2i treatment and was negatively correlated with NPH. Our findings suggest that the anti-albuminuric effect of SGLT2 inhibitors could be attributed to mitigating podocyte apoptosis, attenuating renal fibrosis and promoting weight loss.

## Supplementary Information

Supplementary Information 1.

Supplementary Information 2.

Supplementary Information 3.

Supplementary Information 4.

## Data Availability

All data generated or analyzed during the current study are available from the corresponding author on reasonable request.
