# Supplementary material for: SGLT2 inhibitors attenuate nephrin loss and enhance TGF-β1 secretion in type 2 diabetes patients with albuminuria: a randomized clinical trial
Source: Sci Rep. 2022 Sep 20;12:15695. doi: 10.1038/s41598-022-19988-7 (PMC9489863; doi:10.1038/s41598-022-19988-7)
Supplement: Supplementary file 1 — Supplementary Information 1. [file 41598_2022_19988_MOESM1_ESM.pdf]

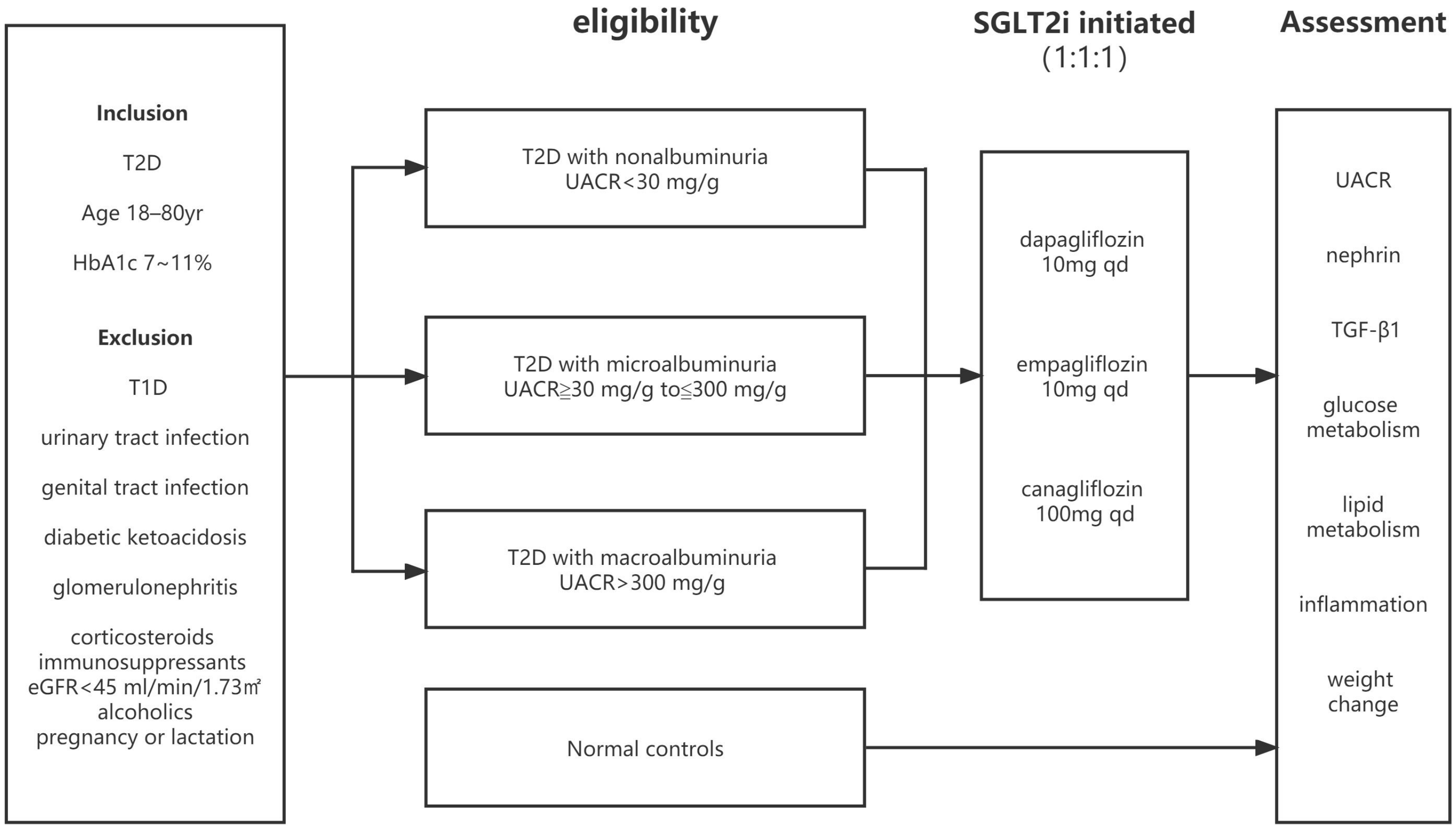

Study Flowchat

T2D：type 2 diabetes;UACR：urinary albumin-to-creatinine ratio；eGFR：estimated glomerular filtration rate
