# Supplementary material for: SGLT2 inhibitors attenuate nephrin loss and enhance TGF-β1 secretion in type 2 diabetes patients with albuminuria: a randomized clinical trial
Source: Sci Rep. 2022 Sep 20;12:15695. doi: 10.1038/s41598-022-19988-7 (PMC9489863; doi:10.1038/s41598-022-19988-7)
Supplement: Supplementary file 2 — Supplementary Information 2. [file 41598_2022_19988_MOESM2_ESM.pdf]

Table S1 Comparisons of proportion of SGLT2 inhibitors initiation in the study

categories	N	Dapagliflozin	Empagliflozin	Canagliflozin	P-value
**UACR<30mg/g**	21	9(42.9%)	5(23.8%)	7(33.3%)	0.254
**UACR⩾30to⩽300mg/g**	20	7(35%)	7(35%)	6(30%)	
**UACR>300mg/g**	27	6(22.2%)	15(55.6%)	6(22.2%)	
**Total**	68	22(32.4%)	27(39.7%)	19(27.9%)	

The data are expressed in number (scale).

Abbreviations:SGLT2: Sodium-glucose cotransporter-2; UACR: urinary albumin-to-creatinine ratio
