# Supplementary material for: SGLT2 inhibitors attenuate nephrin loss and enhance TGF-β1 secretion in type 2 diabetes patients with albuminuria: a randomized clinical trial
Source: Sci Rep. 2022 Sep 20;12:15695. doi: 10.1038/s41598-022-19988-7 (PMC9489863; doi:10.1038/s41598-022-19988-7)
Supplement: Supplementary file 3 — Supplementary Information 3. [file 41598_2022_19988_MOESM3_ESM.pdf]

TabS2 Spearman rank correlations in baseline UACR with NPH, eGFR, Scr, HbA1c, Age, SBP, hsCRP,IL-6

**Variables**	**Correlation coefficients with UACR (*r*)**	***P*-value**
NPH(ug/ml)	0.317	0.005
eGFR(ml/min/1.73m^2)	-0.558	<0.001
Scr(umol/L)	0.299	0.009
HbA1c(%)	0.340	0.003
Age (yrs)	0.398	<0.001
SBP (mmHg)	0.415	<0.001
hsCRP (mg/L)	0.296	0.009
IL-6(pg/ml)	0.260	0.023

Abbreviations: UACR:urine albumin/creatinine ratio : NPH: nephrin eGFR :estimated Glomerular Filtration Rate; Scr: serum creatinine ; HbA_1c:glycated haemoglobin; SBP:Systolic blood pressure; hsCRP :High sensitivity C-reactive protein ;IL-6 Interleukin-6.
