# Supplementary material for: SGLT2 inhibitors attenuate nephrin loss and enhance TGF-β1 secretion in type 2 diabetes patients with albuminuria: a randomized clinical trial
Source: Sci Rep. 2022 Sep 20;12:15695. doi: 10.1038/s41598-022-19988-7 (PMC9489863; doi:10.1038/s41598-022-19988-7)
Supplement: Supplementary file 4 — Supplementary Information 4. [file 41598_2022_19988_MOESM4_ESM.pdf]

TabS3 Spearman rank correlations in NPH with UACR, UTGFβ_1, eGFR, BMI, WC, WHR after 12 weeks SGLT2i treatment in T2D with albuminuria

**Variables**	**Correlation coefficients with NPH (*r*)**	***P*-value**
UACR（mg/g）	0.287	0.006
UTGFβ_1 (pg/ml)	-0.373	<0.001
eGFR(ml/min/1.73m^2)	-0.224	0.034
BMI (kg/m^2)	-0.376	<0.001
WC(cm)	-0.356	<0.001
WHR	-0.248	0.018

Abbreviations: NPH: nephrin; UACR:urine albumin/creatinine ratio; UTGFβ_1:urine transforming-growth-factor-beta$_1$; eGFR :estimated Glomerular Filtration Rate; BMI: body mass index;. WC:Waist Circumference; WHR: Waist hip ratio.
